# Clinical and Translational Significance of Basophils in Patients with Cancer

**DOI:** 10.3390/cells11030438

**Published:** 2022-01-27

**Authors:** Jitesh Chauhan, Chara Stavraka, Melanie Grandits, Lais C. G. F. Palhares, Debra H. Josephs, Katie E. Lacy, James Spicer, Heather J. Bax, Sophia N. Karagiannis

**Affiliations:** 1 St. John’s Institute of Dermatology, School of Basic & Medical Biosciences, King’s College London, London SE1 9RT, UK; jitesh.chauhan@kcl.ac.uk (J.C.); chara.stavraka@kcl.ac.uk (C.S.); melanie.grandits@kcl.ac.uk (M.G.); lais_cristina.palhares@kcl.ac.uk (L.C.G.F.P.); Debra.Josephs@gstt.nhs.uk (D.H.J.); katie.lacy@gstt.nhs.uk (K.E.L.); 2 School of Cancer & Pharmaceutical Sciences, King’s College London, Guy’s Hospital, London SE1 9RT, UK; james.spicer@kcl.ac.uk; 3 Cancer Centre at Guy’s, Guy’s and St Thomas’ NHS Foundation Trust, London SE1 9RT, UK; 4 Breast Cancer Now Research Unit, School of Cancer & Pharmaceutical Sciences, King’s College London, Guy’s Hospital, London SE1 9RT, UK

**Keywords:** basophil, cancer, allergooncology, basophil activation test (BAT), immunotherapy, IgE, type I hypersensitivity, anaphylaxis, survival, gene expression

## Abstract

Despite comprising a very small proportion of circulating blood leukocytes, basophils are potent immune effector cells. The high-affinity receptor for IgE (FcɛRI) is expressed on the basophil cell surface and powerful inflammatory mediators such as histamine, granzyme B, and cytokines are stored in dense cytoplasmic granules, ready to be secreted in response to a range of immune stimuli. Basophils play key roles in eliciting potent effector functions in allergic diseases and type 1 hypersensitivity. Beyond allergies, basophils can be recruited to tissues in chronic and autoimmune inflammation, and in response to parasitic, bacterial, and viral infections. While their activation states and functions can be influenced by Th2-biased inflammatory signals, which are also known features of several tumor types, basophils have received little attention in cancer. Here, we discuss the presence and functional significance of basophils in the circulation of cancer patients and in the tumor microenvironment (TME). Interrogating publicly available datasets, we conduct gene expression analyses to explore basophil signatures and associations with clinical outcomes in several cancers. Furthermore, we assess how basophils can be harnessed to predict hypersensitivity to cancer treatments and to monitor the desensitization of patients to oncology drugs, using assays such as the basophil activation test (BAT).

## 1. Introduction: Characteristics and Functions of Basophils in Protective Immunity, Th2 and Inflammatory Diseases

Basophils are rare circulating granulocytes that make up a relatively small proportion (<1%) of peripheral blood leukocytes. They are characterized by distinctive morphologic features such as irregular cell surface, dense cytoplasmic granules and bilobular nuclei. Basophils express high levels of the tetrameric form (αβγ2) of the high affinity Fc receptor for IgE (FcεRI) on the cell surface. Upon stimulation, either via IgE immune complex formation or by innate signals, basophils can release an array of immune and inflammatory mediators stored in their cytoplasmic granules [1]. Alongside studies in humans, significant research has been undertaken to characterize basophils in other species such as monkeys, rabbits, and mice [2,3,4]. Both mouse and human basophils share many common features—both species express FcεRI and receptors to cytokines such as IL-3, GM-CSF, and IL-33, and can be stimulated to upregulate CD63 and CD203c. This has allowed the use of murine models in the study of basophils and their functions [5,6,7,8,9,10]. Primarily, basophil-depleted mice have been used to investigate the in vivo function of basophils. In some models, monoclonal antibodies targeting FcɛRIα (MAR-1) or CD200R3 (Ba103) have been used to transiently deplete basophils; however, these antibodies can also remove other FcɛRI-expressing cells, such as monocytes, dendritic cells, and mast cells, and Ba103 could activate myeloid and NK cells [11,12]. These models have been used to show that basophils can support Th2 cell differentiation and polarization of adaptive immune responses through secretion of IL-4. Controversially, it has been suggested that basophils may be involved in antigen presenting via MHC class II to CD4^+^ T cells [13,14,15]. More recently, genetically engineered models have allowed both transient [16,17,18,19,20] and long-term ablation [21,22,23] of basophils. Studies in these models have shown that mast cell protease 8 can be used as a differentiation marker in mice, and that basophils are a major source of mast cell protease 11 during parasitic infections. Additionally, it is suggested that basophils may play an essential role in antibody-mediated acquired immunity against ticks [17,19,20]. These have shown that basophils have a role in adaptive immune responses outside of allergy.

Much like other myeloid lineage cells, basophil development derives from hematopoietic stem cells found in the bone marrow through stimulation with IL-3 [24]. The lifespan of a basophil is thought to be relatively short. A study in mice found that the lifespan of basophils could be around 60 hours [25]. Therefore, these cells are continuously generated in the bone marrow and released into the circulation. Despite their low abundance, basophils can be very potent immune cells able to secrete mediators such as granzyme B, TNF-α, and histamine, and are known to play key roles in eliciting powerful effector functions in different allergic diseases, in the manifestation of type 1 hypersensitivity, but also in the immune response to helminth infections.

Conventionally, it has been thought that basophils are largely absent in tissues, except for during certain types of inflammation states such as immune responses to tissue resident parasites [26,27]. There is, however, increasing evidence of the presence and recruitment of basophils in tissues, including in tumor lesions [28]. A study using single-cell RNA sequencing (RNA-seq) has identified the presence of basophils through all stages of normal lung development and provides evidence that these cells may play a larger role in the immune cell network, such as through macrophage imprinting [5]. Recruitment of basophils to inflamed mucosal sites, supported by IL-3, IL-33, and TSLP (thymic stromal lymphopoietin) signals, have been shown in chronic inflammatory bowel diseases, where crosstalk with CD4^+^ memory T cells can promote production of IL-17 and IFNγ [29]. Furthermore, via production of TNF-α, basophils support innate immune responses that confer protection from bacterial infections [30]. Together, these findings suggest a more prominent presence of basophils in all stages of development and in a greater number of anatomical sites beyond the circulation. Therefore, these cells may have wider immunological functions that span beyond Th2 and allergic disorders, such as in inflammation; parasitic, bacterial, and viral infections; and autoimmunity.

Th2-biased inflammation is a well-described feature of many solid tumors. While it is widely appreciated that basophil activation and functions could be heavily influenced by Th2 inflammatory environments and signals, which are known hallmarks of cancer, to date, basophils have received little attention in the cancer context. Hence, in this manuscript, we review the evidence supporting the presence of basophils in patients with solid tumors, and the basophil compartment in the circulation and in cancer lesions. Interrogating publicly available datasets, we conduct gene expression analyses of basophil markers in the tumor microenvironment (TME) and evaluate associations with clinical survival outcomes in several cancer types. Furthermore, we discuss how harnessing attributes of basophils using emerging clinical tools such as the basophil activation test (BAT) can help to monitor desensitization of patients who have shown hypersensitivity to anti-cancer drugs, and to predict whether cancer patients are likely to develop hypersensitivity to emerging therapies, including anti-tumor IgE antibody immunotherapies.

## 2. Basophil Infiltration and Functions in Solid Tumors

Studies have sought to investigate, characterize, and understand the potential functions of basophils in different solid tumor types. Primarily, work has focused on some key tumor types, including melanoma, ovarian, lung, and pancreatic cancers [26,28,31,32].

Several papers have identified basophils recruited to tumors and specifically interacting with the TME in human cancers [31,32,33,34]. In colorectal cancer, low levels of circulating basophils have been found to correlate with higher T stage (size and extent of tumor), higher N stage (number of lymph nodes involved in the vicinity of the tumor), venous and perineural invasion, and overall poorer survival of patients [33]. In mouse models of melanoma, the depletion of regulatory T cells was associated with the infiltration of basophils alongside CD8^+^ T cells into tumor lesions, and CCL3- and CCL4-secreting basophils were shown to promote recruitment of CD8^+^ T cells in the TME. Introduction of IL-3/anti-IL-3 antibody complexes triggered basophilia and infiltration of T cells which were associated with tumor rejection [31].

Research in mouse models has shown that basophils in the TME may exert varying pro-tumorigenic signals, through the release of several Th2 immune mediators such as CD40L, IL-4, IL-6, IL-13, vascular endothelial growth factor (VEGF)-A/B, B cell-activating factor (BAFF), and histamine. Expression of Th2 mediators such as IL-4 and IL-13 are already known to be hallmarks of allergic diseases, but also the balance between expression of mediators such as tumor necrosis factor alpha (TNF-α) with proangiogenic VEGF may point to differential roles in allergy, but also in autoimmune diseases [35,36,37]. However, basophils may also confer anti-tumorigenic properties through the release of pro-inflammatory mediators TNF-α and tryptase [30,38,39,40,41]. Studies in basophil-deficient mouse models suggested important anti-tumor roles and contribution to melanoma rejection, although the opposite was shown in experimental pancreatic cancer models and in patients with pancreatic cancer [31,32]. Overall, these suggest that the role of basophils within the TME will depend on local inflammatory milieu and may vary greatly between cancers.

Crysteinyl leukotrienes (cys-LTs), such as leukotriene C_4_ (LTC_4_), LTD_4_, and LTE_4_, are products of the lipoxygenase pathway of arachidonic acid metabolism. Cys-LTs are known inflammatory lipid mediators in asthma and other inflammatory diseases [42]. These mediators act through distinct CysLT_1_R and CysLT_2_R G-protein-coupled receptors, and through a currently unknown endothelial function they modulate vascular leakage and atherosclerosis in myocardial injury, and retinal angiogenesis [43,44,45,46]. When activated, basophils have been shown to produce LTC_4_ [47,48]. It is suggested that cys-LTs, and in particular LTC_4_, which are produced by human basophils, may be responsible for inducing angiogenesis. A recent study has demonstrated that in CysLT_2_R-deficient mice, tumor challenge resulted in significantly reduced tumor growth, and a smaller number of metastases compared to wild-type mice. Treatment of wild-type mice with a selective CysLT_2_R antagonist reduced tumor volume, vessel density, vascular leakage, and metastases. This suggests that the cys-LT/CysLT_2_R is a VEGF-independent pathway [49]. Basophils can secrete LTC_4_ and VEGF, and thus may contribute to tumor vasculature via a VEGF-independent and VEGF-dependent functions. However, further studies are required to better understand how basophils and their proangiogenic pathways may be targeted and in which tumor types [1]. 

Basophils can produce extracellular DNA traps of mitochondrial DNA and granule proteins. These are also known as basophil extracellular traps (BETs) [50]. Mouse and human basophils can produce BETs, mainly for the purpose of trapping and killing of microbes [51,52]. It has been reported that in inflammatory diseases, upon IL-3 priming and subsequent activation of the compliment 5a receptor or FcεRI, basophils can also produce BETs [50]. Similar research studies focused on neutrophil extracellular traps (NETs) have reported that NETs can favor the development of metastatic disease through further spread and shielding of tumor cells from the host immune cells [53,54,55]. Alongside this, it has been suggested that the NET’s DNA component (NET-DNA) could act as a chemotactic factor that attracts cancer cells via the transmembrane protein CCDC25 expressed on cancer cells, which recognizes extracellular DNA. CCDC25 expression by primary cancer cells has been associated with poor clinical outcomes [56]. These studies highlight that NETs could promote metastasis via a tumor–immune cell interaction mechanism. However, within the current literature, the function of BETs engaging in similar interactions with cancer cells has yet to be elucidated. 

Basophils expressing FcεRI have been implicated in the infiltration and accumulation of IgE in skin following DNA damage and epithelial barrier disruption. Basophil infiltration alongside class-switching and production of endogenous IgE were reported to be protective from epithelial carcinogenesis [57]. In a different study, FoxP3DTR knock-in mouse models were used to deplete the regulatory T cell populations (~99% depletion), provoking a strong infiltration of CD8^+^ T cells and basophils into the TME [31]. Eosinophil-mediated anti-tumoral function, driven through the recruitment of eosinophils by IL-33 into the tumor site, has also been reported [58,59]. A study in asthma has shown that IL-33 upregulated the basophil activation marker, CD63, and an increase in granzyme B production [60]. Similar conditions are possible in some tumor lesions where high IL-33 expression may potentiate an activation state in basophils.

Studies in pancreatic ductal adenocarcinoma (PDAC) patients have identified that the percentage of basophils present in tumor-draining lymph nodes (TDLNs) could be used as an independent prognostic factor following surgery, with higher levels being associated with poorer survival [32]. In the same study, the authors employed a pancreatic cancer mouse model to explore the role of basophils. The recruitment of basophils in TDLNs was found to be influenced by the TME, namely through the release of the leukocyte chemoattractant, CLL7/MCP3, by alternatively activated monocytes. Basophils recruited to the lymph nodes could be activated by T cell-derived IL-3 to release IL-4, which is necessary for Th2 skewing of the immune responses. Notably, in basophil-depleted mice, it was found that the absence of basophils resulted in the absence of the formation of long-term tumors over an 8-week period, in comparison to wild-type mice. These findings suggest that basophils may be required for long-term tumor engraftment [32].

Basophils have been described in lung cancer mouse models as well as in patient lesions. It has been suggested that basophils acquire site-specific cytokine expression profiles due to exposure in the lung microenvironment. In chronic inflammation, exposure of basophils to cytokines, such as IL-33, may trigger basophils to promote the repolarization of lung macrophages to M2-like phenotypes, evident by the expression of the hallmark anti-inflammatory genes (*Arg1, Clec7a, Itgax*) [1,5]. Since alternatively activated M2 macrophages are pro-tumorigenic, these findings mark basophils as potential contributors to tumor inflammation in the lung cancer microenvironment. Single-cell analyses conducted by mass cytometry by time-of-flight (CyTOF), multiplex tissue imaging, and single-cell transcriptomics on early lung adenocarcinoma patient samples pointed to the presence of basophils in both tumor lesions and non-involved lung tissues, although the tumor lesions had significantly lower frequency of basophils compared to non-involved lung tissues, and in the circulation [26]. Human basophils have been shown to release significant levels of IL-4 and IL-13 when co-cultured with lung epithelial adenocarcinoma cells. This activation was mediated by lung adenocarcinoma secreted galactin-3, which bound to IgE on the surface of basophils, to signal their activation in an antigen-independent manner [61,62,63]. Together, these studies may suggest that basophils interact with both immune cells and cancer cells and participate in the inflammation processes observed in lung adenocarcinoma.

Paradoxically, a previous report has suggested that human basophils may be refractory to regulatory T cell (CD4^+^CD25^+^FoxP3^+^)-mediated immunosuppression, and basophils may instead be activated by regulatory T cells [64]. Tumor-draining lymph nodes (TDLNs), also known as sentinel lymph nodes (SLNs), are the first metastatic sites for many primary melanomas and could be the potential sites of immune cell crosstalk. Studies have already sought to characterize NK cells, T cells, dendritic cells, and macrophages [65,66,67,68] within the TDLNs in many cancer types. It has been suggested that there could be a relationship between basophils and B cells, either indirectly via T cells, or orchestrated in conjunction with mast cells through the production of cytokines and co-stimulatory molecules [69]. Basophils stimulated by IgE crosslinked by tumor antigen-expressing cancer cells can produce IL-4 and upregulate CD40L. These are considered classical signals well known to influence B cell signaling and activation and to promote class switching and production of IgE by B cells. These events may occur in tumors and in lymph nodes where T cell help could support B cell maturation and IgE production. However, these interactions have yet to be fully investigated in the context of maintaining health, as well as with regards to their impact on immune responses in malignant diseases.

## 3. Evaluating the Presence and Activation States of Basophils and Associations with Clinical Outcomes in Different Solid Tumors

The studies described above suggest that basophils form part of the immune response in different tumor types. In ovarian cancer, we previously analyzed survival outcomes to ascertain if tumor-infiltrating basophils were positively or negatively associated with patient prognosis within our own ovarian patient cohort, and by analyzing publicly available datasets. In our own cohort of patients with ovarian cancer, we identified that higher numbers of circulating basophils and basophils with greater capacity for ex vivo stimulation were associated with improved survival outcomes. Within larger publicly available datasets, we identified that higher proportions of activated basophils (CCR3, CD123, FcεRI, CD63, CD203c, ± tryptase) signatures in the TME of ovarian cancers were associated with improved progression-free survival (PFS) and overall survival (OS) of patients. This survival benefit was not observed in relation to basophil gene expression signatures alone (CCR3, CD123, FcεRI) [28]. These findings support the presence of basophils within the TME of ovarian cancers, and additionally suggest that activated basophils may have beneficial roles in anti-tumor immunity.

**Figure 1:**
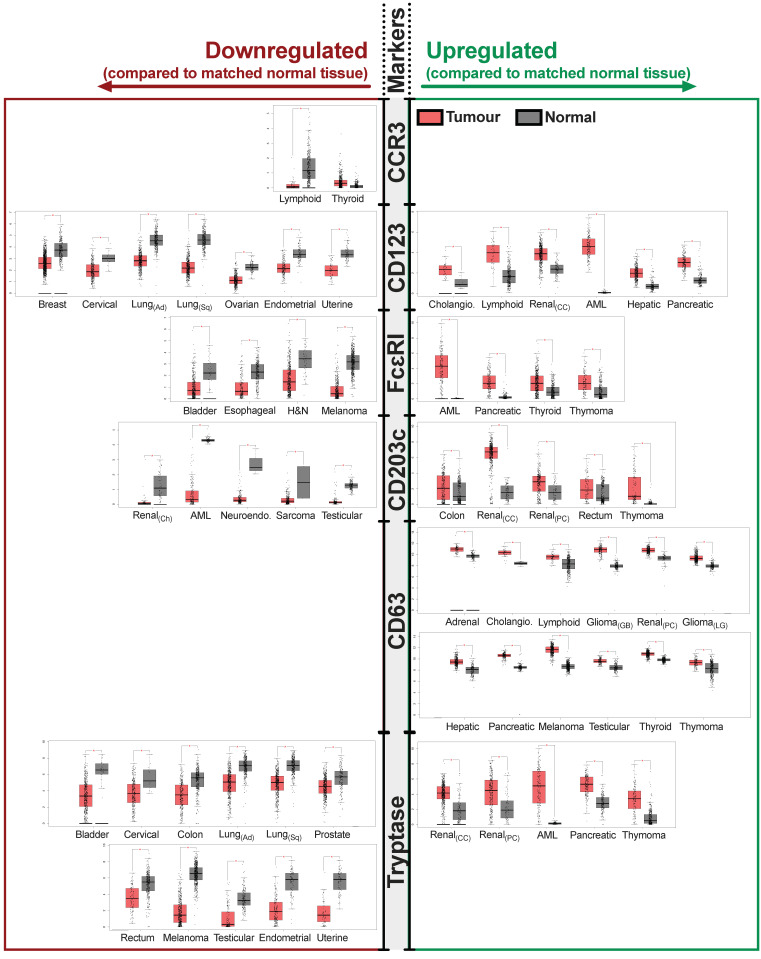
Significant changes in protein expression for basophil markers (CCR3, CD123, FcεRI) and basophil activation markers (CD63, CD203c, tryptase) measured in different tumor types (red) and in the equivalent normal (gray) tissues. Here, we show that in several cancers, there is an upregulation of basophil markers and markers associated with activated basophils, compared to equivalent normal tissues. These may point to the involvement of basophils within some tumor types. Data were obtained from the Gene Expression Profiling Interactive Analysis (GEPIA) online database (http://gepia.cancer-pku.cn/index.html, accessed on 7 December 2021) [70]. Abbreviations and numbers of samples analyzed can be found in Supplementary Table S1. * = *p* ≤ 0.05.

To extend these findings, we sought to evaluate basophil markers in different cancer types and to assess whether gene expression of basophil-associated markers and activated basophil signatures may be associated with clinical outcomes in different cancer types. Gene expression of basophil marker signatures (CCR3, CD123, and FcεRI) and markers of basophil activation (CD63, CD203c, and tryptase) were studied in tumor samples and the equivalent normal tissues in the Gene Expression Profiling Interactive Analysis (GEPIA) online database [70]. We evaluated the gene expression levels for each of the basophil and basophil activation markers associated with the tumor lesions from several cancer types. We summarized data from those cancers that showed a significant up- or down-regulation of a basophil marker when compared to the equivalent normal tissues (Figure 1 and Supplementary Table S1). In these analyses, we found significantly different gene expression profiles of these markers in cancer when compared to equivalent normal tissues from the same site. Gene expression of the basophil marker, CD123, was found to be higher in cholangiocarcinoma, renal, hepatic, and pancreatic cancers. Higher gene expression of the basophil activation markers CD63 and CD203c were detected in several cancers in comparison with normal tissues: cholangiocarcinoma, melanoma, thymoma, glioma, as well as thyroid, renal, hepatic, pancreatic, adrenal, and testicular cancers showed enhanced CD63 expression; increased CD203c was shown in thymoma, colon, renal, and rectum cancers. Although expressed by basophils but also by other immune cells such as mast cells and macrophages in the TME, the high-affinity IgE Fc receptor (FcεRI) was upregulated in thymoma, pancreatic and thyroid cancers. Tryptase was upregulated in both thymoma, pancreatic, and renal cancers. Cholangiocarcinoma, thymoma, renal, and pancreatic cancers appeared to show an overall prominent expression of basophil and activated basophil signatures; for example, pancreatic cancer had upregulated expression of basophil markers, CD123 and FcεRI, and of basophil activation markers, CD63 and tryptase, compared with expression in the corresponding non-malignant tissue.

**Figure 2:**
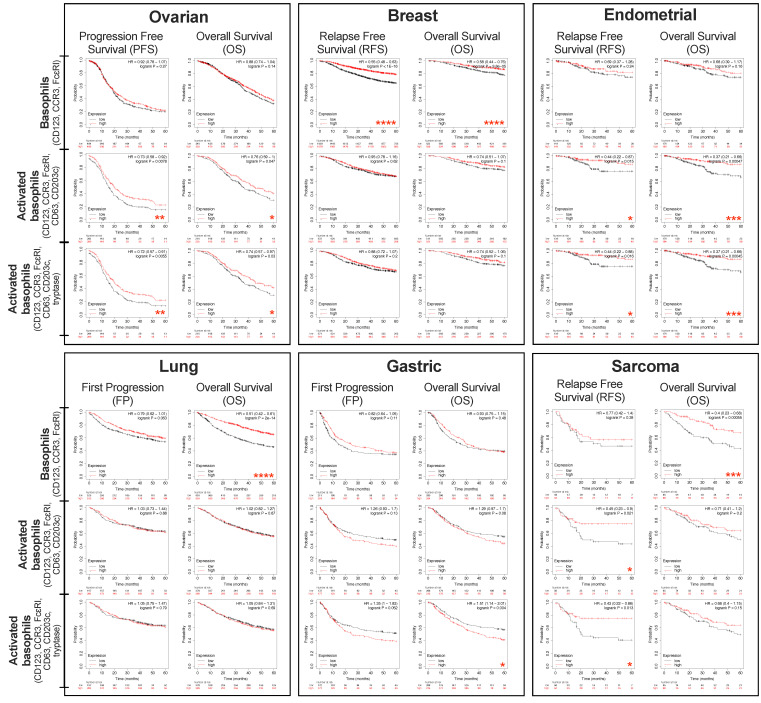
Tumor-resident basophils and associations with cancer patient outcomes. Survival outcome analysis performed using the Kaplan–Meier (KM) Plotter online tool (https://kmplot.com/analysis/, accessed on 7 December 2021) [71]. Gene expression analyses of tumor-resident basophils (CD123, CCR3, and FcεRI) and activated basophil signatures (CD123, CCR3, FcεRI, CD63, CD203c ± tryptase) were performed. Patients’ survival was analyzed up to 60 months. Groups of patients were selected based on combined gene expression levels of selected basophil markers in tumor lesions, grouped into top tertile (T3) and lower tertile (T1), while patients in the middle tertile (T2) were excluded. Markers (Gene Probe ID): CD123 (206148_at); CCR3 (208304_at); FcεRI (211734_s_at); CD63 (200663_at); CD203c (232737_S_at) and tryptase (210084_x_at). Summary of *n* numbers (number of individuals at risk) can be found in Supplementary Table S2. Data sourced through mRNA gene chip (ovarian, breast, lung and gastric) and mRNA RNA-Seq (sarcoma and endometrial). *p* values: * = *p* < 0.05, ** = *p* < 0.01, *** = *p* < 0.001, **** = *p* < 0.0001.

Furthermore, we assessed the potential impact of basophil gene expression in tumor lesions on clinical outcomes in different cancer types (Figure 2 and Supplementary Table S2) using the same basophil and basophil activation marker signatures (Kaplan–Meier (KM) Plotter online tool) [71]. Patients were grouped, based on combined gene expression levels of the selected basophil markers in tumor lesions, into the top tertile (T3), lower tertile (T1), and middle tertile (T2) groups. The T3 group was compared with the T1, while the middle tertile T2 patient group was excluded from our analyses. All plots were analyzed for survival up to 60 months (Figure 2).

Higher expression of basophil markers (CD123, CCR3, and FcεRI) in tumors was associated with significantly better overall survival (OS) in sarcoma and lung cancer. Furthermore, we analyzed basophil markers (CD123, CCR3, and FcεRI) combined with markers of activated basophils (CD63, CD203c, ± tryptase) in relation to patient outcomes. In ovarian cancers, the presence of higher activated basophil signatures (with and without tryptase) was associated with increased progression-free survival (PFS) and OS. In endometrial cancers, the presence of higher activated basophil signatures (with and without tryptase) was associated with increased relapse-free survival (RFS) and OS. In sarcoma, higher levels of activated basophil signatures (with and without tryptase) in the tumor were associated with significantly increased RFS. Conversely, tumors with low basophil activation signatures in gastric cancers were associated with more favorable OS.

**Figure 3:**
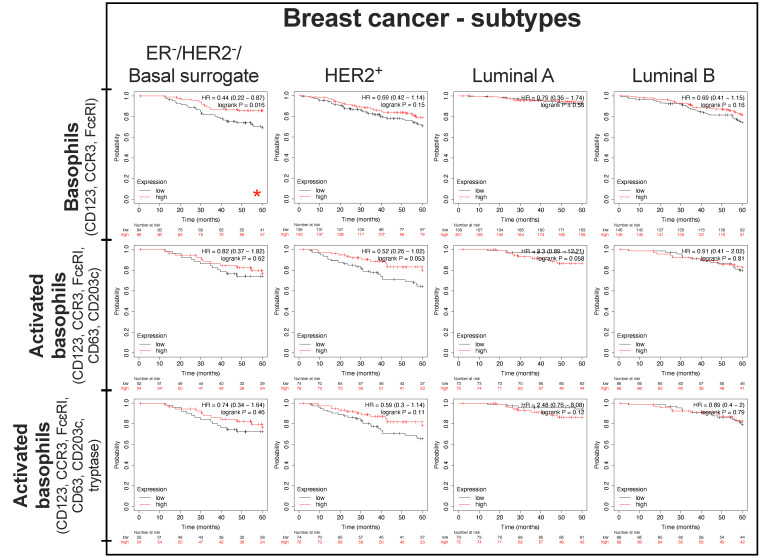
Tumor-resident basophils and clinical outcomes in different breast cancer subtypes. Survival outcome analysis was performed using the Kaplan–Meier (KM) Plotter online tool (https://kmplot.com/analysis/, accessed on 7 December 2021) [71]. Gene expression analyses of tumor-resident basophils (CD123, CCR3, and FcεRI) and activated basophil signatures (CD123, CCR3, FcεRI, CD63, CD203c ± tryptase) were performed. Patient survival outcomes were analyzed over 60 months. Groups of patients were selected based on combined gene expression levels of selected basophil markers in tumor lesions, into top tertile (T3) and lower tertile (T1). Patients in the middle tertile (T2) were excluded. Markers (Gene Probe ID): CD123 (206148_at); CCR3 (208304_at); FcεRI (211734_s_at); CD63 (200663_at); CD203c (232737_S_at) and tryptase (210084_x_at). Summary of *n* numbers (number of individuals at risk) can be found in Supplementary Table S3. Data sourced though mRNA gene chip. *p* values: * = *p* < 0.05.

We previously reported that systemic treatment with antibodies directed to tumor-associated antigens promoted NK cell and macrophage infiltration into tumors in mouse models of breast cancer [72]. Furthermore, we showed bidirectional functional crosstalk between tumor-infiltrating B and T lymphocytes and associations with more favorable overall survival in patients with breast cancer, especially in the highly immunogenic basal breast cancers [73]. Higher expression of basophil markers (CD123, CCR3, and FcεRI) in tumors was associated with significantly better relapse-free survival (RFS) and overall survival (OS) in breast cancer patients. When stratified by breast cancer subtype and observing OS of these patients, higher levels of basophil gene expression signatures were associated with more favorable OS in the ER-/HER2-/basal breast cohort analyzed for survival over 60 months (Figure 3 and Supplementary Table S3).

Overall, gene expression data suggest the presence of basophils and activated basophil signatures, and potential associations of enhanced basophil and basophil activation signatures with more favorable patient outcomes in some tumor types.

## 4. Basophil Activation Studied Ex Vivo to Detect Allergic Reactions to Therapeutic Agents and to Monitor Desensitization to Oncology Drugs

### 4.1. Basophil Activation Testing for the Detection of Allergic Reactions

Basophils play a key role in the IgE-mediated type I hypersensitivity reactions. Their propensity for activation in response to a variety of agents can be evaluated ex vivo using the basophil activation test (BAT), which has been widely utilized for this purpose in the allergy field. The BAT is an emerging assay conducted alongside existing standard of care tests to predict or confirm hypersensitivity, such as the skin prick test and serum β-tryptase levels.

Within the allergy field, the BAT has been applied to detect reactivity to various foods [74,75,76,77,78,79], drugs such as antibiotics [80,81,82,83,84,85,86], and venoms [87,88,89]. This flow cytometric assay is used to identify basophils in unfractionated whole blood, typically by using antibodies for CCR3, CD123, CD203c, and IgE. Basophil activation following the incubation with a positive control, or reactive stimuli, is typically measured by studying upregulation of the basophil activation markers, CD63 or CD203c, on the basophil cell surface, which occurs within a few minutes following stimulation and precedes degranulation. Positive controls in this assay typically activate basophils via IgE-mediated (such as by crosslinking FcεRI) or by innate (such as with the bacterial chemotactic peptide fMLP (*N*-Formylmethionyl-leucyl-phenylalanine)) stimuli [90]. The significant advantage of using whole blood in the BAT is that the presence of allergen-directed IgE antibodies on the basophil cell surface IgE Fc receptors and presence of all potential blood components may more accurately recapitulate the patient setting, and specifically the conditions required for the formation of allergenic stimuli-IgE-FcεRI immune complexes which can trigger basophil activation, degranulation, and the onset of type 1 hypersensitivity. While still used in an experimental setting, in the future, the BAT may become a minimally invasive and useful clinical tool to assess if a therapeutic agent can be administered safely in patients prior to treatment.

### 4.2. Basophil Activation Testing in AllergoOncology

The application of the BAT outside of the allergy field is relatively new. In oncology and within the emerging field of AllergoOncology concerned with the study of IgE and Th2 immune responses in allergy and cancer [91,92], recent studies have sought to utilize the BAT to predict hypersensitivity to a therapeutic agent, such as to chemotherapeutic drugs, and biological agents, such as monoclonal antibodies.

There is evidence that basophils in the circulation of patients with cancer can be activated following administration of chemotherapies, or other monoclonal antibodies such as cetuximab [93,94,95]. The activation of basophils can be measured through the upregulation of cell surface markers on the basophil, caused by the fusion of intracellular vesicles with the cell surface membrane. Cell surface markers such as CD63, CD203c, or both are commonly used to identify the activation of the basophil in the BAT assay. Prior to this work identifying surface markers on basophils, hypersensitivity diagnosis was reliant on clinical history and existing clinical tests, such as skin prick or intradermal test. To confirm hypersensitivity to oxaliplatin, existing clinical assays (skin tests) were used alongside the BAT. It was shown that the BAT could be used as a minimally invasive alternative to screen for hypersensitivity [93]. Likewise, in patients with a history of severe hypersensitivity to carboplatin, and those who have no such history, basophils were incubated with carboplatin and significantly higher levels of FcεRI expression were measured on these patients’ basophils when compared to those from non-hypersensitive patients [94]. This supports an IgE-dependent mechanism participating in carboplatin-induced hypersensitivity.

We have developed a modified version of the BAT which can be applied ex vivo using a small sample of blood from cancer patients—adapted protocol from the commercially available basophil activation test (BAT, Flow2 CAST^®^ kit, Bühlmann Laboratories AG, Switzerland). We have demonstrated that basophils can be identified within whole blood samples taken from cancer patients and can be activated ex vivo to upregulate cell surface CD63 with IgE-mediated (anti-FcεRI, anti-IgE) and with innate (fMLP) stimuli [28,96,97]. We can assess ex vivo if the cancer patients’ basophils can be triggered and upregulate expression of the CD63 cell surface marker when incubated with a therapeutic drug. Although the BAT may offer a chance to study the propensity for type I hypersensitivity to therapeutic agents, a small subset of patients may not benefit from this assay. These include patients who have received prolonged oral corticosteroids, in whom we have observed ablation of basophil populations, and in a small number of “non-responder” patients whose basophils do not respond to any positive control stimuli, and so the results of the assay cannot be interpreted. This “non-responder” population is relatively low in prevalence at 5.8%, consistent with existing literature [28,76,79].

Furthermore, the BAT can be used for monitoring the progress of therapeutic agent desensitization [94,98,99,100,101,102,103,104]. Drug hypersensitivity reactions (DHR) have been on the rise in recent times, which can ultimately restrict the choice of treatment options for patients and negatively impact prognosis. For example, DHR to platinum compound chemotherapies, such as carboplatin, occurs in 9% to 27% of ovarian cancer patients [99,105,106,107]. Drug desensitization involves the administering of incrementally escalating sub-optimal doses of offending drugs with the aim to eventually reach a tolerance to the therapeutic agent [99]. Desensitization is used when (1) no alternative drugs to treat the patient are available, (2) the culprit drug is more effective and associated with fewer side effects, and (3) the culprit drug has a unique mechanism of action which may benefit the patient [108]. Thus, the BAT can play a pivotal role in monitoring the progress of desensitization, and safely identifies the point at which a patient is able to tolerate a dose of the therapeutic agent.

## 5. Basophil Activation Evaluated in the BAT as a Tool to Predict Propensity for Type 1 Hypersensitivity to Emerging IgE Immunotherapy Agents

Of the five antibody classes, only IgG (most often IgG1) has been applied in the clinic for cancer therapy. The use of IgE class therapeutics offers several advantages over the conventional IgG class antibodies presently employed for the treatment of several malignant indications [96]. IgE binds with much greater affinity to its cognate Fc receptors FcεRI and FcεRII/CD23 compared to the affinity of IgGs to their respective Fcγ receptors. High affinity for immune effector cells can translate to prolonged retention and trafficking of the tumor antigen-specific IgE by monocytes, macrophages, and other effector cells to and at the tumor site. This can create localized immune cell activation and release of cytokines, histamine, and other tumor toxic factors which can prevent cancer growth and spread. These attributes of IgE and its ability to activate immune cells towards activated phenotypes and to restrict tumor growth have been investigated in several models of cancer by our group and others [109,110,111,112,113]. The MOv18 IgE trial has hailed the first-in-class IgE antibody, which is tested in a first-in-human phase I clinical trial at Guy’s Hospital, London, and three other trial centers in the U.K. This first-in-class IgE class antibody recognizes folate receptor alpha (FRα), a tumor-associated antigen expressed on a range of cancer types, including ovarian cancer [114].

**Figure 4:**
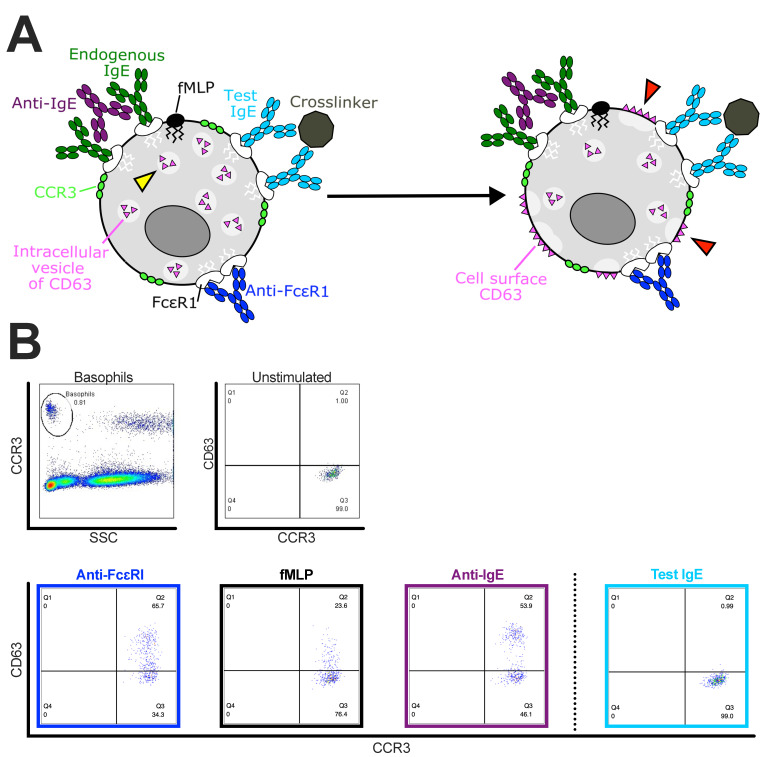
Example of the basophil activation test (BAT) to evaluate the propensity of an anti-FRα IgE antibody (MOv18 IgE) to trigger human basophil activation. (**A**) Schematic of basophil containing intracellular vesicles of CD63 (yellow arrow) and the mechanism of action of stimuli (left). Stimuli include either IgE-mediated (anti-FcεRI or anti-IgE and potential crosslinker of test IgE) or non-IgE-mediated (fMLP). Stimulation of basophils subsequently causes intracellular vesicles of CD63 to fuse with cell surface membrane on the basophil cell surface (red arrows) (right). Basophils can be identified from unfractionated whole blood samples by expression of the CCR3 cell surface marker (left). The change in CD63 surface expression is measured following incubation of basophils with stimuli (right). (**B**) Top: Representative flow cytometric dot plots of human blood cells gated for CCR3^high^SSC^low^ basophil populations (left). Dot plots of unstimulated CCR3^+^ basophils that express low levels of the activation marker CD63 on the cell surface (right). Bottom: When stimulated by one of the three positive control stimuli (anti-FcεRI, navy blue box; fMLP, black box; anti-IgE, purple box) a marked upregulation of CD63-expressing CCR3^+^ basophils is observed. No CD63 expression is observed when the same patient’s blood is incubated with a test IgE (right, light blue box). This indicates that this representative patient would likely not develop hypersensitivity to this test IgE upon intravenous administration of this antibody.

The greatest perceived risk of IgE therapeutics is the potential to trigger basophil degranulation, which can lead to type 1 hypersensitivity and the induction of anaphylaxis. Basophils crosslinked by administered IgE and serum multivalent soluble cancer antigen in patient blood can lead to degranulation and release of histamines and other mediators. If these events occur in the blood, they can potentiate systemic anaphylaxis. Inspired by the emerging studies in the field of allergy, the BAT was developed as one of the experimental assays to be applied alongside several clinical assays for the prediction and monitoring of the potential for anaphylaxis with IgE immunotherapy for the first-in-class IgE in oncology [97,115]. We show in Figure 4A the proposed schema of the mechanism of action of three positive controls (anti-FcεRI, fMLP, and anti-IgE) in the upregulation of cell surface CD63 through the fusion of intracellular vesicles with the cell surface membrane in the accompanying representative flow plots of the upregulation of basophil activation marker CD63 in CCR3^+^ basophils (Figure 4B). We also show the proposed mechanism for crosslinking of the test IgE, and the accompanying representative plot, showing that there is no such activation of basophils caused by the IgE (Figure 4A,B). Prior to clinical testing, the BAT was applied to evaluate the propensity of MOv18 IgE to activate basophils from patients with ovarian cancer, the primary malignant indication for folate receptor-α-targeted treatments. FRα shed from the FRα-positive tumors is thought to be monovalent, and thus is unable to crosslink MOv18 IgE bound on the surface of basophils. However, we hypothesize that MOv18 IgE could be crosslinked in certain conditions. Perhaps a polyvalent form of the FRα could be shed, or the combination of shed monovalent FRα and the production of autoantibodies against FRα could cause crosslinking of the MOv18 IgE on the cell surface and trigger basophil activation. Basophils in 41 out of 42 of the patients studied were not activated when incubated ex vivo with MOv18 IgE. This was irrespective of prior treatment history of the patient, of the total IgE, serum tryptase levels, and regardless of FRα cancer antigen expression in tumors, circulating FRα, or anti-FRα autoantibodies [97].

Preliminary data from the MOv18 IgE trial have shown that an IgE can safely be administered systemically in patients with cancer, whilst mitigating the risk of the most common perceived toxicity risk for IgE immunotherapy, systemic anaphylaxis [114].

## 6. Conclusions

For many years, basophils have been clearly identified to have a role in allergy. However, the wider role of basophils in other disorders has not been fully explored and thus not sufficiently understood. Significantly, there is now increasing appreciation that basophils may have roles outside allergic responses, and more recent work is starting to explore these elusive cells in different cancers. However, controversies surrounding a role of basophils in antigen presentation underline the need for further research into their functions outside of allergic responses. Specifically in cancer, studies using different animal models have reported contradicting findings for the roles of basophils in the TME. Some genetically engineered models show that basophils exert pro-tumorigenic functions [31,116]. Conversely, in a different model using a monoclonal antibody to deplete basophils, the authors concluded that basophils have anti-tumor properties [31]. This highlights that data in mouse models should be interpreted tentatively. There is an unmet need for an animal model in which only basophils are depleted, and in which all basophils are ablated, a combination that current models have not achieved. Overall, it is unclear if any of these studies accurately represent the contributions of these cells in human malignancy. Further work is required to understand the role of cysteinyl leukotrienes (cys-LTs) and basophil extracellular traps (BET) in relation to basophils in the TME of primary cancer lesions and in metastatic sites. Triggers of autophagy and ferroptosis are being increasingly found to contribute to cancer; however, the specific role of basophils in these processes has yet to be investigated. By being recruited to different anatomical sites throughout tissue development and to tumor lesions, basophils may be involved in immune cell imprinting of macrophages and, alongside other cells, contribute to the rejection of some tumors. However, there is still a large gap in our understanding of the specific mechanisms by which basophils function and their interactions with other immune cells. The anatomical locations of these interactions may be the circulation and tumor-draining lymph nodes (TDLNs), but there is a lack of understanding of how these short-lived cells traffic in and out of different anatomical sites and the dynamics of their interactions with tumor cells and immune cells.

Through our own analysis of publicly available datasets, we have shown the presence of basophil and activated basophil markers within a range of tumors (Figure 1). Through these analyses, we have also shown that for sarcoma, lung, and breast cancer, higher numbers of basophils are beneficial for survival outcomes. When evaluating activated basophil gene expression signatures, this survival benefit extends to sarcoma, endometrial, and ovarian cancers. However, this may not be true for all cancers, as our analyses showed that lower levels of basophil gene expression signatures were associated with more favorable survival in gastric cancer (Figure 2). These indicate that similarly to other immune cells, basophils may be influenced by the local inflammatory milieu and thus confer juxtaposing pro-tumor or anti-tumor roles in different tumor microenvironments.

Our previous work on IgE-based anti-cancer antibodies has shown that basophils are key IgE cells which can be interrogated to assess the safety of IgE immunotherapy in patients with cancer. We have previously shown that circulating basophils from patients with ovarian cancer are able to respond to IgE-mediated and non-IgE-mediated signals, and that the basophil activation test (BAT) can identify hypersensitivities to a range of therapeutic agents, including chemotherapeutic agents [117,118]. These findings suggest that basophils retain their ability to be activated in cancer and may participate in the induction of hypersensitivity to anti-cancer drugs. The assessment of the safety of IgE is still lacking and further work is required to gain a clearer understanding of basophils and their contributions to anti-cancer immunity. Through an adapted version of the BAT, we have sought to investigate this further throughout the first-in-man trial of MOv18 IgE, where we have successfully conducted the BAT as a companion to the pre-clinical assessment and clinical testing (Figure 4). Application of the BAT may help evaluate the propensity for type 1 hypersensitivity and mitigate the perceived risk of administering monoclonal IgE class antibodies directed against tumor-associated antigens as therapeutic agents in cancer patients. Our work regarding the BAT and MOv18 IgE could be replicated for all IgE therapeutic candidates in the future. The propensity of basophils from cancer patients to be activated by non-IgE signals and by IgE antibodies both in the clinical testing of IgE immunotherapies and in the pre-clinical development of novel IgE antibodies has not been extensively undertaken in larger patient cohorts. Subsequently, studies to understand the mechanisms of action of anti-tumor IgE and its propensity for triggering basophil activation are still required in the pre-clinical setting in sufficient patient cohorts to provide an early indication of safety.

In conclusion, the understanding of basophil biology outside of the allergic response has progressed significantly in recent times. However, elucidating the roles of basophils in human immunity is still needed, and particularly in cancer, where increasing evidence suggests the potential participation of basophils in immune cell and cancer cell crosstalk and priming of other cell types. Furthermore, there is much more to understand about basophils with the introduction of IgE-based therapeutic agents in clinical testing, where basophils may be harnessed in assessing safety and to support the development of novel and more effective treatments for cancer.

## Data Availability

Protein gene expression data obtained from the Gene Expression Profiling Interactive Analysis (GEPIA) online database (http://gepia.cancer-pku.cn/index.html, accessed on 7 December 2021) [70]. Survival outcome analysis performed using the Kaplan–Meier (KM) Plotter online tool (https://kmplot.com/analysis/, accessed on 7 December 2021) [71].

## Supplementary Materials

The following are available online at https://www.mdpi.com/article/10.3390/cells11030438/s1: Supplementary Table S1: Protein expression abbreviation and sample numbers (GEPIA online database); Supplementary Table S2: Summary of Figure 2 *n* numbers (number at risk); Supplementary Table S3: Summary of Figure 3 *n* numbers (number at risk).
