# Supplementary material for: Clinical and Translational Significance of Basophils in Patients with Cancer
Source: Cells. 2022 Jan 27;11(3):438. doi: 10.3390/cells11030438 (PMC8833920; doi:10.3390/cells11030438)
Supplement: Supplementary file 1 [file cells-11-00438-s001.zip › cells-1522193-supplementary.pdf]

Supplementary Table S1: Summary of basophil-associated markers, tumor and normal tissue samples studied for levels of gene expression by interrogating the Gene Expression Profiling Interactive Analysis (GEPIA) online database (http://gepia.cancer-pku.cn/index.html).

Gene marker	Tumor type	Abbreviation	Number of tumor samples	Number of normal samples
CCR3	Lymphoid	-	47	337
	Thyroid	-	512	337
CD123	Breast	-	1085	291
	Cervical	-	306	13
	Lung adenocarcinoma	$Lung_{(Ad)}$	483	347
	Lung squamous cell carcinoma	$Lung_{(Sq)}$	486	338
	Ovarian	-	426	88
	Endometrial	-	174	91
	Uterine	-	57	78
	Cholangiocarcinoma	Cholangio.	36	9
	Lymphoid	-	47	337
	Kidney renal clear cell carcinoma	$Renal_{(CC)}$	523	100
	Acute myeloid leukemia	AML	173	70
	Hepatic	-	369	160
	Pancreatic	-	179	171
FcεRI	Bladder	-	404	28
	Esophageal	-	182	286
	Head and Neck	H&N	519	44
	Melanoma	-	461	558
	Acute myeloid leukemia	AML	173	70
	Pancreatic	-	179	171
	Thyroid	-	512	337
	Thymoma	-	118	339
CD203c	Kidney chromophobe	$Renal_{(Ch)}$	66	53
	Acute myeloid leukemia	AML	173	70
	Neuroendocrine	Neuroendo.	182	3
	Sarcoma	-	262	2
	Testicular	-	137	165
	Colon	-	275	349
	Kidney renal clear cell carcinoma	$Renal_{(CC)}$	523	100
	Kidney renal papillary cell carcinoma	$Renal_{(PC)}$	286	60
	Rectum	-	92	318
	Thymoma	-	118	339
CD63	Adrenal	-	77	128
	Cholangiocarcinoma	Cholangio.	36	9
	Lymphoid	-	47	337
	Glioblastoma	$Glioma_{(GB)}$	163	207
	Kidney renal papillary cell carcinoma	$Renal_{(PC)}$	286	60
	Lower grade glioma	$Glioma_{(LG)}$	518	207
	Hepatic	-	369	160
	Pancreatic	-	179	171

	Melanoma	-	461	558
	Testicular	-	137	165
	Thyroid	-	512	337
	Thymoma	-	118	339
Tryptase	Bladder	-	404	28
	Cervical	-	306	13
	Colon	-	275	349
	Lung adenocarcinoma	Lung(Ad)	483	347
	Lung squamous cell carcinoma	Lung(Sq)	486	338
	Prostate	-	492	152
	Rectum	-	92	318
	Melanoma	-	461	558
	Testicular	-	137	165
	Endometrial	-	174	91
	Uterine	-	57	78
	Kidney renal clear cell carcinoma	Renal(CC)	523	100
	Kidney renal papillary cell carcinoma	Renal(PC)	286	60
	Acute myeloid leukemia	AML	173	70
	Pancreatic	-	179	171
	Thymoma	-	118	339

Supplementary Table S2: Summary of Figure 2 *n* numbers (number at risk).

Cancer	Group	Survival outcome	Expression level	Time (months)						
				0	10	20	30	40	50	60
Ovarian	Basophils (CD123, CCR3, FcεRI)	Progression Free Survival (PFS)	Low	484	340	187	104	67	42	34
			High	486	360	211	141	105	70	48
		Overall Survival (OS)	Low	546	456	370	274	186	129	92
			High	553	479	386	294	214	146	98
	Activated basophils (CD123, CCR3, FcεRI, CD63, CD203c)	Progression Free Survival (PFS)	Low	203	143	68	40	29	17	14
			High	209	149	84	53	35	18	11
		Overall Survival (OS)	Low	216	198	159	111	76	54	41
			High	223	196	153	114	77	51	35
	Activated basophils (CD123, CCR3, FcεRI, CD63, CD203c, tryptase)	Progression Free Survival (PFS)	Low	204	144	67	39	28	16	13
			High	209	149	83	52	34	18	11
		Overall Survival (OS)	Low	216	198	159	111	76	54	41
			High	223	195	153	113	77	50	35
Breast	Basophils (CD123, CCR3, FcεRI)	Relapse Free Survival (RFS)	Low	1630	1493	1315	1157	999	877	756
			High	1670	1601	1470	1313	1170	1022	882
		Overall Survival (OS)	Low	623	603	569	518	463	421	361
			High	638	624	600	564	517	488	431
	Activated basophils (CD123, CCR3, FcεRI, CD63, CD203c)	Relapse Free Survival (RFS)	Low	672	626	541	483	411	362	305
			High	691	638	576	501	438	373	298
		Overall Survival (OS)	Low	311	305	285	258	230	210	182
			High	321	312	296	266	240	205	172
	Activated basophils (CD123, CCR3, FcεRI, CD63, CD203c, tryptase)	Relapse Free Survival (RFS)	Low	671	621	536	475	405	355	295
			High	691	639	582	504	445	388	317
		Overall Survival (OS)	Low	311	305	285	258	227	206	176
			High	321	312	296	266	239	206	174
Endometrial	Basophils (CD123, CCR3, FcεRI)	Relapse Free Survival (RFS)	Low	141	126	95	72	49	39	28
			High	143	125	98	67	48	40	36
		Overall Survival (OS)	Low	179	154	122	97	62	44	34
			High	183	164	129	95	71	58	51
	Activated basophils (CD123, CCR3, FcεRI, CD63, CD203c)	Relapse Free Survival (RFS)	Low	139	126	90	54	30	25	18
			High	143	134	106	83	66	55	46
		Overall Survival (OS)	Low	179	158	118	85	45	35	25
			High	184	167	134	102	83	70	60
	Activated basophils (CD123, CCR3, FcεRI, CD63, CD203c, tryptase)	Relapse Free Survival (RFS)	Low	139	126	90	54	30	25	18
			High	143	134	105	81	65	55	46
		Overall Survival (OS)	Low	179	158	118	85	45	35	25
			High	184	167	134	103	83	70	60
Lung	Basophils (CD123, CCR3, FcεRI)	First Progression (FP)	Low	325	256	212	185	155	119	89
			High	332	290	254	216	178	140	104
		Overall Survival (OS)	Low	636	505	419	353	297	256	215
			High	653	596	545	479	399	322	253
	Activated basophils (CD123, CCR3, FcεRI, CD63, CD203c)	First Progression (FP)	Low	197	167	146	131	107	75	53
			High	203	173	146	126	103	81	64
		Overall Survival (OS)	Low	378	337	291	249	203	163	123
			High	389	344	296	253	218	189	163
	Activated basophils (CD123, CCR3, FcεRI, CD63, CD203c, tryptase)	First Progression (FP)	Low	197	168	147	132	107	74	52
			High	203	172	146	126	104	82	65
		Overall Survival (OS)	Low	378	338	295	254	208	166	125
			High	389	347	297	255	220	193	167
Gastric	Basophils	First Progression (FP)	Low	211	109	76	63	58	53	37
			High	218	148	107	89	78	62	54

	(CD123, CCR3, FcεRI)	Overall Survival (OS)	Low	289	200	151	121	106	102	86
			High	294	243	185	142	120	106	86
	Activated basophils (CD123, CCR3, FcεRI, CD63, CD203c)	First Progression (FP)	Low	172	119	93	82	76	62	45
			High	177	123	81	68	60	52	45
		Overall Survival (OS)	Low	208	174	145	122	113	108	90
			High	215	176	137	113	96	85	68
	Activated basophils (CD123, CCR3, FcεRI, CD63, CD203c, tryptase)	First Progression (FP)	Low	172	118	94	84	76	63	45
			High	177	123	83	69	62	53	46
		Overall Survival (OS)	Low	208	175	147	126	117	113	94
			High	215	171	130	106	88	78	60
Sarcoma	Basophils (CD123, CCR3, FcεRI)	Relapse Free Survival (RFS)	Low	50	37	20	15	12	10	7
			High	52	40	26	21	17	15	11
		Overall Survival (OS)	Low	86	69	47	38	24	19	14
			High	88	79	64	53	35	29	22
	Activated basophils (CD123, CCR3, FcεRI, CD63, CD203c)	Relapse Free Survival (RFS)	Low	50	39	23	16	14	12	8
			High	52	39	28	21	17	15	12
		Overall Survival (OS)	Low	85	74	56	43	32	29	21
			High	88	76	55	42	30	24	21
	Activated basophils (CD123, CCR3, FcεRI, CD63, CD203c, tryptase)	Relapse Free Survival (RFS)	Low	50	39	22	15	13	11	7
			High	52	39	28	21	17	15	12
		Overall Survival (OS)	Low	85	74	57	44	32	29	21
			High	88	76	57	44	31	25	21

Supplementary Table S3: Summary of Figure 3 *n* numbers (number at risk).

Breast - subtype	Group	Expression level	Time (months)						
			**0**	**10**	**20**	**30**	**40**	**50**	**60**
$ER^-/HER2^-$/Basal surrogate	Basophils (CD123, CCR3, FcεRI)	Low	84	83	76	69	62	52	41
		High	86	86	82	79	72	66	57
	Activated basophils (CD123, CCR3, FcεRI, CD63, CD203c)	Low	52	51	49	44	40	33	29
		High	54	54	50	47	42	36	24
	Activated basophils (CD123, CCR3, FcεRI, CD63, CD203c, tryptase)	Low	52	51	49	43	39	32	28
		High	54	54	50	47	42	36	24
$HER2^+$	Basophils (CD123, CCR3, FcεRI)	Low	139	131	121	105	86	77	67
		High	143	137	128	117	107	99	79
	Activated basophils (CD123, CCR3, FcεRI, CD63, CD203c)	Low	74	70	64	57	46	43	37
		High	76	73	70	59	51	41	33
	Activated basophils (CD123, CCR3, FcεRI, CD63, CD203c, tryptase)	Low	74	70	65	57	45	41	37
		High	76	73	69	58	50	40	33
Luminal A	Basophils (CD123, CCR3, FcεRI)	Low	199	197	194	186	180	171	163
		High	201	198	195	184	174	168	158
	Activated basophils (CD123, CCR3, FcεRI, CD63, CD203c)	Low	73	73	72	70	65	65	62
		High	75	74	71	63	57	49	44
	Activated basophils (CD123, CCR3, FcεRI, CD63, CD203c, tryptase)	Low	73	73	72	69	65	65	61
		High	75	74	71	62	57	48	43
Luminal B	Basophils (CD123, CCR3, FcεRI)	Low	73	73	72	69	65	65	61
		High	75	74	71	62	57	48	43
	Activated basophils (CD123, CCR3, FcεRI, CD63, CD203c)	Low	66	65	65	62	57	55	46
		High	68	66	63	60	56	48	41
	Activated basophils (CD123, CCR3, FcεRI, CD63, CD203c, tryptase)	Low	66	65	65	62	58	54	44
		High	68	66	64	60	56	49	42
